# The Thermometer in Trephining for Epilepsy

**Published:** 1880-05

**Authors:** 


### THE THERMOMETER IN TREPHINING FOR EPILEPSY.

ACCORDING to the *Broca*, in deciding the question of trephining in epilepsy from convulsions, etc., occurring in a patient who has a depression of the skull resulting from a previous fall or blow, the thermometer will settle our doubts. If the temperature is raised on the injured side, the effects are due to the lesion, and the depressed fragment must be raised; in the contrary case, the symptoms are not due to the lesion, and operative interference is contra-indicated.—*Medical Record.*

